# Biofilms: The Microbial “Protective Clothing” in Extreme Environments

**DOI:** 10.3390/ijms20143423

**Published:** 2019-07-12

**Authors:** Wen Yin, Yiting Wang, Lu Liu, Jin He

**Affiliations:** State Key Laboratory of Agricultural Microbiology, College of Life Science and Technology, Huazhong Agricultural University, Wuhan 430070, China

**Keywords:** microorganism, extreme environment, biofilm, extracellular polymeric substances (EPS), adaptative mechanism

## Abstract

Microbial biofilms are communities of aggregated microbial cells embedded in a self-produced matrix of extracellular polymeric substances (EPS). Biofilms are recalcitrant to extreme environments, and can protect microorganisms from ultraviolet (UV) radiation, extreme temperature, extreme pH, high salinity, high pressure, poor nutrients, antibiotics, etc., by acting as “protective clothing”. In recent years, research works on biofilms have been mainly focused on biofilm-associated infections and strategies for combating microbial biofilms. In this review, we focus instead on the contemporary perspectives of biofilm formation in extreme environments, and describe the fundamental roles of biofilm in protecting microbial exposure to extreme environmental stresses and the regulatory factors involved in biofilm formation. Understanding the mechanisms of biofilm formation in extreme environments is essential for the employment of beneficial microorganisms and prevention of harmful microorganisms.

## 1. Introduction

In the natural world, there exist some extremely harsh environments [1,2], such as high-intensity ultraviolet (UV) radiation, high or low temperature, high alkalinity, high acidity, high salinity, high pressure, poor nutrients, plentiful antibiotics, etc. Previously, these environments are considered to be life prohibiting; however, after further exploration, these extreme environments are in fact found to contain abundant microorganisms. They are not only alive, but also thrive well in extreme environments that were formerly thought to be inhospitable to life [3]. Microorganisms that are able to survive in these extreme environments are called extremophiles [4,5], which include radiation-resistant extremophiles, thermophiles, psychrophiles, alkaliphiles, acidophiles, halophiles, piezophiles, etc. Each microorganism surviving under these extreme conditions exhibits its specific resistant mechanism, among which the role of biofilm is considered to be crucial [6,7].

A biofilm is an organized aggregate of microorganisms living within a self-produced matrix of extracellular polymeric substances (EPS) that is attached to a biotic or abiotic surface [8,9,10]. It is considered to be one of the most widely distributed and successful modes of life on the Earth [11], and in most natural environments, a biofilm is the predominating microbial lifestyle [12]. Indeed, fossilized biofilms have been found in a 3.2 billion-year-old deep-sea volcanogenic massive sulfide deposit from the Pilbara Craton of Australia [13], and in a 3.3 to 3.4 billion-year-old hydrothermally influenced sediment from the Barberton greenstone belt of South Africa [14]. Similar biofilm structures have also been identified in modern hydrothermal systems, such as deep-sea vents [15] and hot springs [16]. These data suggest that the ability to form biofilms can protect microorganisms from extreme environments [17,18]. Further studies have also shown that biofilms can increase microbial resistances against UV radiation [19], extreme temperature and pH [20,21,22], high salinity [23], high pressure [24], poor nutrients [25], various antibiotics [26], etc. The resistance of biofilms against extreme environments seems to be capable of creating a suitable habitat for microbial populations, makes the exchange of substances and information between microorganisms more convenient, and is thus a self-protective mechanism for the growth of microorganisms [27]. The morphological structures of microorganisms in the biofilm and their sensitivity to environmental factors as well as biological characteristics are substantially different to planktonic microorganisms [28]. Such a three-dimensional structure of biofilm seems to provide a natural barrier and protective layer to microorganisms; in addition, the immobile microorganisms in EPS are more resistant to extreme environments than planktonic microorganisms [29].

In the current review, we try to provide contemporary perspectives on the molecular mechanism of biofilm formation, the roles of biofilm in extreme environments, and the various signaling cascades involved in biofilm formation. It aims at providing new ideas for the employment of beneficial microorganisms, as well as the prevention and control of harmful microorganisms.

## 2. Microbial Biofilms

Biofilm formation is a complex and dynamic process, in which organized communities of microorganisms are encased in a matrix of EPS that cluster microbial cells together [30,31]. EPS are mainly composed of polysaccharides, proteins, lipids, and nucleic acids (RNA and extracellular DNA (eDNA)) [32,33], which form a highly hydrated polar mixture that contributes to the overall scaffold and three-dimensional structure of a biofilm.

**Figure 1:**
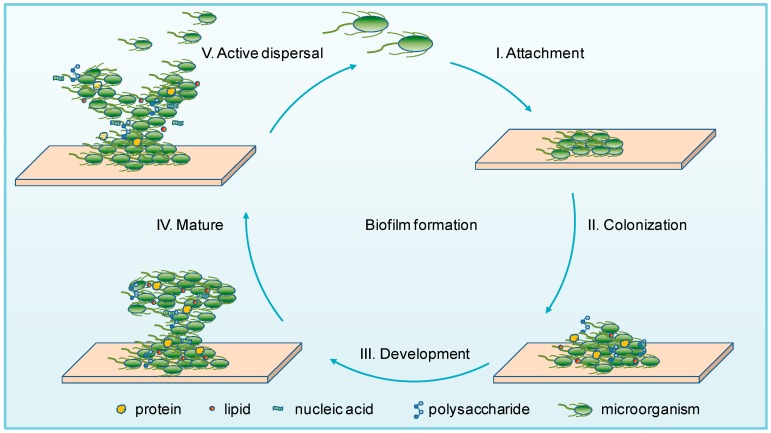
Model of microbial biofilm formation. Biofilm formation consists of five distinct stages: I. Attachment: microbial cells adhere to the surface reversibly. II. Colonization: microbial cells attach to the surface irreversibly via flagella, pili, exopolysaccharides, etc. III. Development: multilayered cells accumulate and produce extracellular polymeric substances (EPS). IV. Mature: stable formation of a three-dimensional community. V. Active dispersal: microorganisms are disseminated from the aggregate biofilm and return to a planktonic state.

The biofilm lifestyle is an endless cycle, and the process of biofilm formation can be summarized into the following five major stages based on the previous studies [34,35,36] (Figure 1): (I) Attachment: microorganisms are reversibly adsorbed to a surface via weak interactions (such as the van der Waals forces) with a biotic or abiotic surface [37]; (II) Colonization: microorganisms are irreversibly attached to the surface via stronger hydrophilic/hydrophobic interactions by flagella, pili, lipopolysaccharides, exopolysaccharides, collagen-binding adhesive proteins, etc. [38,39]; (III) Development: the multilayered cells are accumulated by proliferation, and EPS are produced and secreted [32,40]; (IV) Maturation: stable formation of a three-dimensional community that contains channels to effectively distribute nutrients and signaling molecules within the biofilm [41]; (V) Active dispersal: microbial cells are detached in clumps or separated, due to interactions with either intrinsic or extrinsic factors, with the disseminated cells subsequently colonizing on other locations [42].

In this dynamic process, specific enzymes are involved in degrading and reconfiguring biofilm, resulting not only in partial matrix degradation, but also the active dispersal of biofilm and subsequent surface recolonization [7]. Attachment is the beginning for a biofilm formation, while active dispersal is not the end, but also the creation of the next round of biofilm formation. The continuous recirculation of biofilm gives the microorganisms the ability to adapt to various extreme environments.

## 3. Biofilms in Extreme Environments

**Figure 2:**
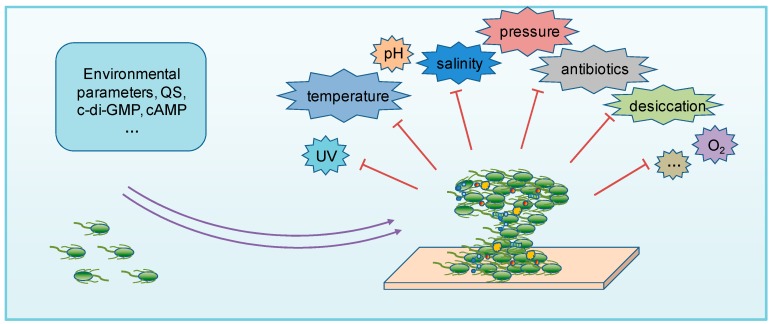
A schematic representation of the biofilm function. Biofilm formation can increase the resistances of microorganisms to various extreme environments.

Biofilm formation is a unique growth mode chosen by microorganisms in response to various environmental stresses, and previous studies have shown that the ability to form biofilms is important for microorganisms to grow in various extreme environments (Figure 2).

### 3.1. Biofilm in UV Radiation

The solar UV radiation comprises three types based on the range of wavelength: UV-A (320 to 400 nm), UV-B (290 to 320 nm), and UV-C (100 to 290 nm) [43]. Besides their implication in damaging proteins and membranes, UV-A can damage DNA by generating reactive oxygen species to induce single-strand DNA breaks [44,45], while UV-B can be absorbed directly by DNA to alter or mutate nucleotides [46]. On the other hand, UV-C is the most energetic source that produces more photoproducts than either UV-A or UA-B radiation [47].

RM4440 is a *Pseudomonas aeruginosa* FRD1 derivative that carries a plasmid-based *recA*-*luxCDABE* fusion that serves as a *Pseudomonas aeruginosa* whole-cell biosensor for monitoring DNA damage [48]. Elasir et al. have immobilized RM4440 in an alginate matrix to simulate the biofilm formation to study the response of biofilm to UV radiation damage [49]. The results reveal that compared to planktonic bacteria, the matrix of EPS seems to be protective in physically shielding microorganisms against UV-C, UV-B, and UV-A radiations, and transmitting only 13%, 31%, and 33% of the UV light, respectively, to the microorganisms. Thus, biofilms are effective at protecting microbial cells from UV radiation and exposure.

Biofilm formation can also protect *Listeria monocytogenes* from UV-C radiation [50]. *Listeria monocytogenes* N53-1 that was isolated from salmon smoke house and allowed to form a biofilm for seven days exhibited higher UV-C resistance than that incubated for only one hour. Recently, Enyedi et al. revealed that there is a high microbial diversity in the biofilms of naturally radioactive hydrothermal spring caves, which had been especially adapted to an environment of high radioactivity of the subsurface [51]. Also, they found that the higher radioactivity, the less diversity but more radiation-resistant microbial communities in biofilms.

*Deinococcus geothermalis* is a representative of the extremely radiation-resistant family of *Deinococcaceae* [52]. Frösler et al. demonstrated that the biofilm of *Deinococcus geothermalis* DSM 11300 appears to be more UV-tolerant than that of planktonic cells, and speculated that it is possibly due to the generation of reactive oxygen species from the photodissociation of water molecules retained in the cells or matrix of EPS in the biofilm [53].

Biofilm formation is one of clever strategies for microorganisms to survive UV exposure. The study of mechanisms of biofilm formation under UV radiation is also helpful for the control of harmful microorganisms. A number of studies have shown that with the protection of biofilms by “radiation-resistant clothing”, a variety of microorganisms are more active under extreme UV radiation environments. Thus, a better understanding of the microbial-resistant mechanism to UV may help provide protection for the human aerospace industry.

### 3.2. Biofilm in Extreme Temperature

Temperature exerts a significant influence on microorganisms, and biofilm can adequately explain the effects of microorganisms against extreme temperatures. Cihan et al. studied the biofilm formation of thermophilic bacteria in the *Bacillaceae* family at different temperatures, and revealed that an incubation temperature at 65 °C is more effective in biofilm production than at 55 °C for the *Thermolongibacillus*, *Aeribacillus*, *Geobacillus*, and *Anoxybacillus* thermophilic genera [54].

Some species in *Sulfolobus* genus are thermophilic acidophiles that grow optimally at 75 °C [55,56]. Koerdt et al. tested the biofilm formation at temperatures ranging from 60 to 85 °C, and the results demonstrate that the amounts of biofilm in both *Sulfolobus acidocaldarius* (isolated from Yellowstone National Park, United States (USA)) and *Sulfolobus solfataricus* (a European isolate from Italy) are increased at 60 °C and 85 °C. At 60 °C, they showed a fivefold and fourfold increase in biofilm formation, respectively [17].

Studies of cold-adapted bacterium *Bacteriovorax* showed that at temperatures below 10 °C, the number of bacteria is significantly reduced in the water column, but not in the surface biofilms [57]. Further studies by Williams et al. showed that at 5 °C, *Bacteriovorax* lives 50% longer in biofilms than in suspension [58]. Also, EPS in biofilm from Antarctic bacteria (cold-tolerant *Winogradskyella* CAL384 and CAL396, psychrophiles *Colwellia* GW185, and *Shewanella* CAL606) showed an ability to form stable emulsions to protect cells from freeze–thawing cycles, thus increasing the adaptability of microbial cells to cold environments [59].

Therefore, biofilm formation enables microorganisms in extreme environments to become more resistant to damage caused by temperature stress. Throughout environmental changes, biofilm can act as a “protective clothing” to provide a suitable habitat for their survival and metabolism. In the extreme temperature environment, the biofilm serves more as a “smart garment” when dealing with such high temperatures: it can resist the external high temperature and render the interior suitable for growth and reproduction. On the other hand, biofilm can also stabilize the internal environment when it is extremely cold outside, causing no freeze of the cells and enabling them to survive.

### 3.3. Biofilm in Extreme pH Environments

Biofilms also help microorganisms resist the effects of extreme pH, in which both acidophiles and alkaliphiles generally exist in biofilms [60,61,62].

Species abundance usually decreases under extremely acidic conditions, but a large number of acidophiles that are protected in biofilm are still prevalent [63]. In fact, under extremely acidic pH, it has been found that the solubility of heavy metals increase, and therefore increasing the toxicity index. The combination of extremely acidic pH and heavy metals together was found to lead to a significant change in the EPS composition of the biofilm, which plays a vital role in the adaptation of microorganisms to extreme environments [3]. It can not only prevent heavy metal toxicity, but also capture and enrich trace elements [64]. Besides, the inositol and 3-O-methylglucose contents in EPS were found to positively correlate with the toxicity index. Early research showed that inositol polyphosphates can inhibit the formation of hydroxyl radicals by ferric iron, which can decrease its toxicity [65], and methylglucose-containing polysaccharides from marine microorganisms have also been exploited to remove heavy metals from solutions [66]. This data suggests that biofilms play a protective effect in extremely acidic environments that are mediated, at least in part, by specific sugars.

Under alkaline conditions, the alkaliphilic communities have also been shown to form biofilms to enclose the microorganisms in a matrix of EPS [67,68]. Charles et al. showed that under a thick EPS layer, *Alishewanella* and *Dietzia* are capable of growth at pH values between 11.0–11.5, and maintain internal pH values of 10.4 and 10.7, respectively, demonstrating that biofilm formation can provide a shelter for alkaliphilic communities to survive hyperalkaline stress to a large extent [69].

To compare the responses of bacteria in biofilm or planktonic state to alkaline stress, clinical isolates of Enterococcus faecalis, Lactobacillus paracasei, Olsenella uli, Streptococcus anginosus, Streptococcus gordonii, Streptococcus oralis, and Fusobacterium nucleatum from infected root canals were placed under a stress at pH 10.5 for 4 h. The results showed that bacteria can survive alkaline shifts better in the biofilm than in the planktonic state [70]. Additionally, van der Waal et al. reported that increased EPS production in biofilm protects *Enterococcus faecalis* against 20 mM of Ca(OH)_2_ [71].

In general, microorganisms shielded in biofilms can resist extreme pH stress better than in a planktonic state. However, the exact mechanisms behind the survival of microorganisms in biofilms exposed to acidic and alkaline challenge is worthy of further studies. As an acid-resistant and alkali-resistant “strong protective clothing”, it is still unclear whether the biofilm is correlated with the biological evolution of bacteria. With further exploration, it will be interesting to discover the characteristic features and structure of this “protective clothing”.

### 3.4. Biofilm in High-Salinity Environments

There are many halophiles on the Earth, most of which are found in high-salinity environments, such as salt lakes, marine environments, and inland saline soils. In these places, microbial cytoplasmic lysis and cell death are significantly caused by increasing osmotic pressure [72,73]. Microorganisms can, in fact, form biofilms that are resistant to high salt damage [74,75]. Indeed, Amjres et al. isolated a halophilic strain *Halomonas stenophila* HK30 from a saline wetland in Brikcha (Morocco) that is able to form biofilm in a medium with 5% *w*/*v* salt [76]. Mallic et al. showed that the two halophiles of *Kocuria flava* AB402 and *Bacillus vietnamensis* AB403, which were isolated from mangrove rhizosphere of Sundarban, can not only form biofilms effectively, but also produce a large amount of EPS under salt stress; they can also use EPS to develop inherent resistance and adsorb a large amount of metal elements, etc. [77].

Many researchers have conducted a variety of salt tolerance tests of non-halophiles to study their resistant mechanisms. Kim et al. evaluated the effects of salinity on the biofilm formation of *Vibrio* sp. B2 isolated from seawater, brine, and a biofouled membrane coupon, and found that the salinity-stressed bacteria still maintain good cellular activity and overproduce EPS, which exhibits a high potential to cause biofouling and biofilm formation [23]. Zhao et al. studied the composition of EPS from microbial biofilms at different salinity, and found that the production of both proteins and polysaccharides contents of soluble, loosely, or tightly bound EPS in biofilm increase with increasing salinity [78].

EPS are the main components of the biofilm, which acts as a gel-like matrix that binds cells together to form aggregates and provide protection for microorganisms against high salt stress [79]. Whether halophiles or non-halophiles, the biofilm produced by them may thus play an important role in agricultural productions, because they can be used to adsorb various metal elements to help crop growth and promote soil bioremediation under salt stress.

### 3.5. Biofilm in High-Pressure Environments

Piezophiles (barophiles) are microorganisms whose survival and reproduction is optimized to high pressures, such as those in deep-sea environments. Since the piezophiles are difficult to separate and culture, and live with restrict distribution [80], there have been very few studies on their biofilm formation. A few available studies only show that an increase in pressure leads to an increase in the expression of the outer membrane protein [81]. To make up for the deficiency of the research on biofilm formation of piezophiles, the research on the biofilm of non-piezophiles under high pressure is increasing.

High hydrostatic pressure (HHP) can especially alter many macromolecules in microorganisms and also affect their translation and transcription within the cells, leading to the production of dysfunctional proteins [82,83]. Studies on HHP biofilms have shown that microorganisms in the biofilms are more resistant to high pressure than planktonic microorganisms. Also, biofilms formed by Gram-negative bacteria are more resistant to HHP than those of Gram-positive bacteria [82].

Biofilm formation can also enhance microbial tolerance to high mechanical pressure. Hou et al. compared the responses of biofilms to mechanical pressure between an EPS-producing *Staphylococcus aureus* ATCC 12600 and a non-EPS-producing *Staphylococcus aureus* 5298, and found that the biofilm of former bacterium exhibits a higher resistance to exerted mechanical force because it can yield an immediate increase in polysaccharide content [24].

Under high-pressure stimulation, the “pressure-resistant garments” of both piezophiles and non-piezophiles become more “solid”. The studies of formation mechanism and structural composition of the “pressure-resistant garments” in high-pressure environments will thus play important roles in the fields of medicine, industry, and biotechnology.

### 3.6. Biofilm in Oligotrophic Conditions

Under oligotrophic conditions, microbial growth may be affected due to poor nutrients. However, during this time, biofilm formation is also found to play a role in the microbial tolerance to the restricted of microbial growth.

Bacteria can be separated into two general groups regarding the nutrient requirement in the living environments: copiotrophs and oligotrophs, which grow optimally in high and low nutrient conditions, respectively.

For copiotrophs, some authors concluded that biofilm production is enhanced in the poor nutrient medium [84,85]. For example, Cherifi et al. investigated the biofilm formation of copiotroph *Listeria monocytogenes* (isolated from pork slaughterhouses and cutting facilities) under a rich medium (brain heart infusion, BHI) or a 10-fold diluted BHI (BHI/10) [86]. They demonstrated that the biofilm biovolume in BHI/10 is significantly higher than that in BHI. Further investigation revealed that relative poor nutrients enhance cell death and release eDNA, leading to enhanced biofilm formation and structural stability. *Vibrio cholerae* is also a special kind of copiotrophs; when placed in an environment lacking glucose and mannose, *Vibrio cholerae* A1552 seems to stimulate cyclic adenosine monophosphate (cAMP) synthesis, to form a cAMP–cAMP receptor protein (CRP) complex to regulate the expression of downstream genes related to nutrient acquisition and utilization, leading to the stimulation of biofilm formation [87,88].

Oligotrophs are the dominant strains in oligotrophic environments and are more common in pure water. As oligotrophs, the non-tuberculous mycobacteria are the natural inhabitants in pure water or engineered water systems and soils, and are able to grow at low carbon concentrations [89]. During its growth, biofilms help oligotrophs resist the oligotrophic environments. Although there are few available studies on oligotrophic biofilms, it is not difficult to speculate that under oligotrophic conditions, biofilm formation is a good survival strategy for oligotrophs.

Under oligotrophic conditions, such “protective clothing” takes a series of measures to ensure the normal reproduction and metabolism of microorganisms, and preferentially distributes limited nutrients within this “protective clothing” [90]. Thus, biofilms seem to play a huge role in the survival of bacteria in the extreme oligotrophic conditions. Regardless of their types, biofilms act as a “protective garment” for microorganisms, and are responsible for protecting their survival and reproduction.

### 3.7. Tolerance and Resistance to Antibiotics in Biofilms

Microorganisms in biofilms seem to show a strong tolerance and resistance to antibiotics. Microbial tolerance is generally related to the mode of biofilm formation, which is a transient and nonheritable phenotype [91]. Microbial resistance is an acquired ability of microorganisms to resist antibiotics in an inheritable mode [92]. Several molecular mechanisms are involved in the biofilm-specific tolerance and resistance.

Biofilms can confer microbial tolerance to antibiotics mainly through the following types [26,93].

First, biofilms can serve as physical barriers, and their thickness and chemical composition can prevent the perfusion of antibiotics [94]. There are many anionic and cationic molecules in the EPS of biofilms, such as uronic acids, proteins, glycoproteins, glycolipids, eDNA, etc. They can also bind to charged antibiotics and form a shelter for microorganisms [40,95], in order to help microbial cells embedded within the biofilms establish tolerance against antibiotics [96]. Singh et al. demonstrated that the penetration of oxacillin, cefotaxime, and vancomycin are significantly reduced through the biofilms of *Staphylococcus aureus* and *Staphylococcus epidermidis* [97]. The adsorption of antibiotics by biofilm components [98] or the degradation of antibiotics by hydrolase, such as a β-lactamase [99,100], can also limit antibiotic penetration. Pel exopolysaccharide, which is cross-linked with negatively charged eDNA in the *Pseudomonas aeruginosa* matrix of EPS [101], can also play both structural and protective roles to reduce its susceptibility to aminoglycoside antibiotics [102].

Second, physiological limitations including the growth rate [103,104,105], biofilm age [106], starvation [107], etc., can reduce biofilm susceptibility to the antibiotics. Williamson et al. demonstrated that the subpopulation of inactive bacteria harbored in *Pseudomonas aeruginosa* biofilms is resistant to killing by tobramycin and ciprofloxacin, but the actively growing population remains sensitive to antibiotic killing [108]. Besides, a small subpopulation of bacteria in biofilm and persister cells entering a slow-growing or starving state is also highly tolerant to killing by antibiotics [109].

Microorganisms growing in biofilms are more resistant to antibiotics than in planktonic counterparts. Several authors have also revealed an interconnection between biofilm formation and the resistance to antibiotics [110,111].

First, microbial genetic diversification in biofilms can contribute to resistances to antibiotics [112]. Second, the biofilm is considered to be a main reservoir of genetic diversity that promotes microbial survival in extreme environments, leading to the development of resistances to antibiotics. It is reported that biofilm formation causes an increasing average plasmid copy number as well as the increasing transcription of plasmid-borne resistant genes in *Enterococcus faecalis* cells [113]. This finding suggested that biofilm growth can reduce microbial susceptibility to antibiotics.

Second, multidrug efflux pumps in biofilms can transport antibiotics to prevent toxic accumulation [114,115,116]. The PA1874-1877 efflux pump in *Pseudomonas aeruginosa* is more expressed in the biofilm state than in the planktonic state, and is involved in resistance to antibiotics [117]. Deletion of the PA1874 to PA1877 genes encoding this pump in *Pseudomonas aeruginosa* PA14 increases the microbial sensitivity to tobramycin, gentamicin, and ciprofloxacin, especially when this mutant strain is present in a biofilm.

Third, sub-minimal inhibitory concentrations (sub-MICs) of antibiotics can also induce resistances to antibiotics. In clinical isolates of *Staphylococcus epidermidis*, sub-MICs of erythromycin [118], tetracycline, and quinupristin–dalfopristin [119] seem to enhance the expression of intercellular adhesion gene clusters, leading to increased EPS expression and invasiveness. Sub-MICs of β-lactam antibiotics also stimulate a thicker biofilm via the upregulated genes that are involved in glycogen biosynthesis in the non-typeable *Haemophilus influenzae* strains that were isolated from patients with chronic otitis and chronic bronchitis [120].

Furthermore, polymicrobial biofilms show enhanced resistances to antibiotics [121,122,123]. For example, *Candida albicans*, β-1,3-glucan, can bind with ofloxacin, and *Escherichia coli* cells embedded within the *Candida albicans* biofilm show increased resistance to ofloxacin compared to the monomicrobial *Escherichia coli* biofilm [124]. Also, polymicrobial biofilms formed by *Staphylococcus albicans* and *Candida albicans* are often found in different types of infections, with *Staphylococcus albicans* coated in the matrix secreted by *Candida albicans* showing enhanced resistance to vancomycin [125,126,127].

Relatively speaking, the studies of “protective clothing” of microorganisms are more thorough and comprehensive in the fields of tolerance and resistance to antibiotics than other resistant mechanisms, but people are paying more attention to learning how to destroy or take off this “protective clothing” to reduce the damage that it causes to humans.

Together, the biofilm can provide a shelter for microorganisms to survive against many kinds of extreme environments. In addition to those discussed above, biofilms can also protect microorganisms from several types of acute environmental stressors such as desiccation, heavy metal pollution, oxidative stress, etc. [3,128] (Figure 2). For example, space habitats living in the International Space Station (ISS) are under extreme UV radiation, desiccation, temperature, and pressure stresses, but it was demonstrated that these stresses can trigger abundant EPS production and biofilm formation. The Mir space station was found to be heavily colonized by biofilms, which damaged quartz windows and corroded various metal surfaces [129]. As studies on environmental biofilms in extreme habitats are rare, further investigation into the mechanisms regulating the biofilm formation of microorganisms, especially those of the environmental isolates (and environmental isolates in situ) in response to different stresses is important. Understanding the structure and protection mechanism of this versatile and magical “protective clothing” in extreme environments is not only a scientific study, but also benefits human life, since it will be a good way for production services.

## 4. Regulation of Biofilms in Extreme Environments

The emergence of biofilms in extreme environments is the result of a series of changes in gene expression. It is a struggle for microbial survival to resist extreme environments by regulating the expression of some genes. In extreme environments, the formation, composition, and function of biofilms seem to be inseparable from a series of regulatory systems.

### 4.1. Quorum Sensing-Based Signaling

Quorum sensing (QS) is a group behavior in which microorganisms regulate their gene expression profile according to the changes of cell density in a population. It is an induction phenomenon that occurs when the number of microorganisms reaches a certain density threshold. The QS system of microorganisms is mainly involved in the differentiation of microbial biofilms (Table 1), and is considered to be an indispensable part of microbial transmission mechanisms in extreme environments [2]. In response to various extreme conditions, QS seems to play important roles in the regulation of biofilm formation [130,131].

In extreme environmental responses, QS can regulate more than 10% of genes in *Pseudomonas aeruginosa* that are primarily implicated in the production of virulence factors, biofilm formation, resistances to antibiotics, mortality, and the amendment of metabolic pathways [132]. In fact, QS is involved in the tolerance of *Pseudomonas aeruginosa* biofilms to kanamycin, tobramycin, and hydrogen peroxide [133,134]. This data may be related to the role of QS in the eDNA production regulation, which inhibits the penetration of some antibiotics into biofilm [135]. *Vibrio cholerae* can pass through the acidic stomach alive before reaching the upper intestine, by developing a thick, glutinous biofilm mediated by the QS [136]. The *Vibrio cholerae* mutant that is deficient in the QS regulator, HapR, which inhibits the expression of the *Vibrio* polysaccharide biosynthesis operon, seems to exhibit a thicker biofilm [136]. However, when the *Vibrio cholerae* comes out of the acidic environment of the stomach, biofilm protection is not further required, and HapR instead facilitates the disruption of the biofilm [137].

In extremophiles, there are three primary QS systems: autoinducer-1 (AI-1), AI-2, and peptide-based. The AI-1 system is found to be more prevalent (except in the thermophiles that favor AI-2), while the peptide-based system was the least abundant in extremophiles. The AI-1 system uses N-acyl-homoserine lactones (AHLs) as signals [142], and is associated with the biofilm formation in *Acinetobacter baumannii* and *Pseudomonas aeruginosa* [138]. AI-2, a furanosyl borate diester, is shared by both Gram-positive and Gram-negative bacteria. For example, AI-2 regulates the biofilm formation in *Bifidobacterium longum* [139]; meanwhile, AI-2 not only regulates biofilm formation, but also enhances anti-oxidative stress in *Deinococcus radiodurans* [143]. In acidophiles, approximately 4.5% genes of *Acidithiobacillus ferrooxidans* ATCC 23270 T account for the genes involved in the QS network, of which 42.5% are used for biofilm formation regulation. Thus, one may presume that QS regulates biofilm formation in this bacteria via a complex signal system [144].

### 4.2. Nucleotide Second Messenger-Based Signaling

Nucleotides such as cyclic dimeric guanosine 3′,5′-monophosphate (c-di-GMP), cyclic adenosine 3′,5′-monophosphate (cAMP), etc., are critical elements of the signal transduction networks, which link perceptions of the environment to the specific cellular behaviors of prokaryotes (Table 1). These molecular mechanisms are particularly crucial in microorganisms that are exposed to certain types of extreme environments [145].

c-di-GMP is a novel and ubiquitous bacterial second messenger that was shown to control biofilm formation in response to environmental influence [146,147,148]. It has been reported that EPS components such as polysaccharides, flagella, pili, adhesins, and eDNA contribute to biofilm formation and are regulated by c-di-GMP through specific receptors [149,150,151]. Generally, an increased concentration of intracellular c-di-GMP promotes surface attachment and biofilm formation, while decreased intracellular c-di-GMP concentration induces biofilm dispersal [152] (Table 1). In some acidophiles, c-di-GMP signaling is related to biofilm formation [145]. For example, *Acidithiobacillus* can thrive in extreme environments with poor nutrients, high concentrations of heavy metals, and extreme acidity. Indeed, several PilZ-containing receptors of *Acidithiobacillus* are found to be related with Type IV pilus formation and twitching motility, which are implicated in the irreversible attachment to surfaces, microcolony grouping, and the structural development of biofilms [153,154]. The *pel*-like operon, encoding the c-di-GMP receptor protein PelD, is also found in the *Acidithiobacillus* strains. A recent report demonstrated that PelD and the c-di-GMP signal pathway are involved in the biofilm formation and structure in *Acidithiobacillus thioooxidans* [140].

Another bacterial second messenger, cAMP, is also found in a variety of prokaryotes and seems to be conserved among diverse bacteria. Huynh et al. demonstrated that cAMP is involved in the dispersal of biofilms in *Pseudomonas aeruginosa* [141] (Table 1). Also, cAMP concentration can control the biofilm production of *Vibrio cholerae* [88] (Table 1). In addition, Paytubi et al. demonstrated that cAMP is involved in the spatial distribution to modulate biofilm locations [155]. In a rich medium with low osmolarity at 25–28 °C and static incubation, *Salmonella* can form a biofilm at the air–liquid interface, which is known as the pellicle, while in the minimal media, a solid–liquid interface biofilm called bottom biofilm is observed [155].

As nucleotide second messengers, c-di-GMP and cAMP respond to various environmental signals and regulate biofilm formation through various mechanisms [156]. The research on signal regulation system can help reveal the mechanism of biofilm formation and provide a direct target for biofilm prevention and control.

## 5. Conclusions

In extreme environments, microorganisms regulate the expression of a series of biofilm-forming genes through QS, nucleotide second messenger-based signaling, etc., to endow microorganisms with the capability of becoming resistant to various extreme environments such as UV radiation, extreme temperature and pH, high salinity, high pressure, poor nutrients, antibiotics, etc.

Protection by microbial biofilms seems also to play an essential role in the production of special enzyme preparations for the pharmaceutical industry, food industry, agricultural production, environmental protection, energy utilization, and other fields of industry, as well as scientific research. Biofilms have several protective advantages that may be physical, physiological, or genetic. An in-depth study of the protective mechanism of biofilms to microorganisms in extreme environments is expected to resolve contradictions between the extreme environments of industrial production and the limited stability of enzyme proteins, allowing one to establish a high efficiency and low-cost bioprocessing technology. Further, microorganisms in biofilms display different features to those in planktonic states, and this peculiar form of development endows associated microorganisms with a high tolerance to extreme environments.

The current review provides comprehensive information on the biofilm formation, biofilms in extreme environments, and biofilm regulation, which may provoke new strategies for the utilization and treatment of biofilms in extreme environments.

## Figures and Tables

**Table 1:** Main affecting factors in biofilm formation.

	Factor	Brief Description	Strain	Reference
Environmental parameter	Ultraviolet (UV)	Matrix of extracellular polymeric substances (EPS) shows a protective property by physical shielding against UV radiation	*Listeria monocytogenes, Deinococcus geothermalis*	[51,53]
	Temperature	Biofilm formation increases at high temperature, and the composition of biofilms change at low temperature. They both enhance microbial resistance	*Thermolongibacillus, Sulfolobus*, etc.	[17,54]
	Extreme pH	At extreme pH, biofilms increase bacterial resistance by altering EPS content	*Enterococcus faecalis*, *Alishewanella*, etc.	[69,70]
	Salinity	Bacteria can withstand high salt damage by aggregating into granular biofilm forms	*Halomonas stenophila, Kocuria flava* AB402, etc.	[76,77]
	High pressure	High pressure yield an immediate increase in the polysaccharide band area of bacterial biofilms	*Staphylococcus aureus*	[24]
	Poor nutrient	Under poor nutrient conditions, the biofilm formation is enhanced	*Listeria monocytogenes*, non-tuberculous mycobacteria	[86,89]
Quorum sensing (QS)	Autoinducer-1 (AI-1)	AI-1 system is related to biofilm formation, and can adjust its amount in extreme environments	*Acinetobacter baumannii*, *Pseudomonas aeruginosa*	[138]
	Autoinducer-2 (AI-2)	AI-2 regulates biofilm formation against environmental stresses	*Bifidobacterium longum*	[139]
Messenger molecule	cyclic dimeric guanosine 3’,5’-monophosphate (c-di-GMP)	c-di-GMP can control biofilm formation in response to different environments	*Acidithiobacillus thioooxidans*	[140]
	cyclic adenosine 3’,5’-monophosphate (cAMP)	cAMP is crucial for the formation of pellicle biofilm	*Pseudomonas aeruginosa* *,* *Vibrio cholerae*	[88,141]

## References

[1] Rothschild L.J., Mancinelli R.L. (2001). Life in extreme environments. Nature.

[2] Kaur A., Capalash N., Sharma P. (2019). Communication mechanisms in extremophiles: Exploring their existence and industrial applications. Microbiol. Res..

[3] Blanco Y., Rivas L.A., Gonzalez-Toril E., Ruiz-Bermejo M., Moreno-Paz M., Parro V., Palacin A., Aguilera A., Puente-Sanchez F. (2019). Environmental parameters, and not phylogeny, determine the composition of extracellular polymeric substances in microbial mats from extreme environments. Sci. Total Environ..

[4] Gabani P., Singh O.V. (2013). Radiation-resistant extremophiles and their potential in biotechnology and therapeutics. Appl. Microbiol. Biotechnol..

[5] Wakai S. (2019). Biochemical and thermodynamic analyses of energy conversion in extremophiles. Biosci. Biotechnol. Biochem..

[6] Frols S. (2013). Archaeal biofilms: Widespread and complex. Biochem. Soc. Trans..

[7] Flemming H.C., Wingender J., Szewzyk U., Steinberg P., Rice S.A., Kjelleberg S. (2016). Biofilms: An emergent form of bacterial life. Nat. Rev. Microbiol..

[8] Huang H., Peng C., Peng P., Lin Y., Zhang X., Ren H. (2018). Towards the biofilm characterization and regulation in biological wastewater treatment. Appl. Microbiol. Biotechnol..

[9] Hoiby N. (2014). A personal history of research on microbial biofilms and biofilm infections. Pathog. Dis..

[10] Gupta P., Sarkar S., Das B., Bhattacharjee S., Tribedi P. (2016). Biofilm, pathogenesis and prevention—A journey to break the wall: A review. Arch. Microbiol..

[11] Stoodley P., Sauer K., Davies D.G., Costerton J.W. (2002). Biofilms as complex differentiated communities. Annu. Rev. Microbiol..

[12] Watnick P., Kolter R. (2000). Biofilm, city of microbes. J. Bacteriol..

[13] Rasmussen B. (2000). Filamentous microfossils in a 3235-million-year-old volcanogenic massive sulphide deposit. Nature.

[14] Westall F., de Wit M.J., Dann J., van der Gaast S., de Ronde C.E.J., Gerneke D. (2001). Early Archean fossil bacteria and biofilms in hydrothermally-influenced sediments from the Barberton greenstone belt, South Africa. Precambrian Res..

[15] Taylor C.D., Wirsen C.O., Gaill F. (1999). Rapid microbial production of filamentous sulfur mats at hydrothermal vents. Appl. Environ. Microbiol..

[16] Reysenbach A.L., Cady S.L. (2001). Microbiology of ancient and modern hydrothermal systems. Trends Microbiol..

[17] Koerdt A., Godeke J., Berger J., Thormann K.M., Albers S.V. (2010). Crenarchaeal biofilm formation under extreme conditions. PLoS ONE.

[18] Rinaudi L.V., Giordano W. (2010). An integrated view of biofilm formation in rhizobia. FEMS Microbiol. Lett..

[19] De Carvalho C.C.C.R. (2017). Biofilms: Microbial strategies for surviving UV exposure. Adv. Exp. Med. Biol..

[20] Harrison J.J., Ceri H., Turner R.J. (2007). Multimetal resistance and tolerance in microbial biofilms. Nat. Rev. Microbiol..

[21] Norwood D.E., Gilmour A. (2001). The differential adherence capabilities of two *Listeria monocytogenes* strains in monoculture and multispecies biofilms as a function of temperature. Lett. Appl. Microbiol..

[22] Hostacká A., Ciznár I., Stefkovicová M. (2010). Temperature and pH affect the production of bacterial biofilm. Folia Microbiol..

[23] Kim L.H., Chong T.H. (2017). Physiological responses of salinity-stressed *Vibrio* sp. and the effect on the biofilm formation on a nanofiltration membrane. Environ. Sci. Technol..

[24] Hou J., Veeregowda D.H., van de Belt-Gritter B., Busscher H.J., van der Mei H.C. (2018). Extracellular polymeric matrix production and relaxation under fluid shear and mechanical pressure in *Staphylococcus aureus* biofilms. Appl. Environ. Microbiol..

[25] Marsden A.E., Grudzinski K., Ondrey J.M., DeLoney-Marino C.R., Visick K.L. (2017). Impact of salt and nutrient content on biofilm formation by *Vibrio fischeri*. PLoS ONE.

[26] Hathroubi S., Mekni M.A., Domenico P., Nguyen D., Jacques M. (2017). Biofilms: Microbial shelters against antibiotics. Microb. Drug Resist..

[27] Chen Y., Yan F., Chai Y., Liu H., Kolter R., Losick R., Guo J.H. (2013). Biocontrol of tomato wilt disease by *Bacillus subtilis* isolates from natural environments depends on conserved genes mediating biofilm formation. Environ. Microbiol..

[28] Lewis K. (2010). Persister cells. Annu. Rev. Microbiol..

[29] Van Houdt R., Michiels C.W. (2010). Biofilm formation and the food industry, a focus on the bacterial outer surface. J. Appl. Microbiol..

[30] Donlan R.M. (2002). Biofilms: Microbial life on surfaces. Emerg. Infect. Dis..

[31] Renner L.D., Weibel D.B. (2011). Physicochemical regulation of biofilm formation. MRS Bull..

[32] Flemming H.C., Wingender J. (2010). The biofilm matrix. Nat. Rev. Microbiol..

[33] Powell L.C., Pritchard M.F., Ferguson E.L., Powell K.A., Patel S.U., Rye P.D., Sakellakou S.M., Buurma N.J., Brilliant C.D., Copping J.M. (2018). Targeted disruption of the extracellular polymeric network of *Pseudomonas aeruginosa* biofilms by alginate oligosaccharides. NPJ Biofilms Microbi..

[34] Sadekuzzaman M., Yang S., Mizan M.F.R., Ha S.D. (2015). Current and recent advanced strategies for combating biofilms. Compr. Rev. Food Sci. Food Saf..

[35] Sharahi J.Y., Azimi T., Shariati A., Safari H., Tehrani M.K., Hashemi A. (2019). Advanced strategies for combating bacterial biofilms. J. Cell Physiol..

[36] Rasamiravaka T., Labtani Q., Duez P., EI Jaziri M. (2015). The formation of biofilms by *Pseudomonas aeruginosa*: A review of the natural and synthetic compounds interfering with control mechanisms. BioMed Res. Int..

[37] Bos R., van der Mei H.C., Busscher H.J. (1999). Physico-chemistry of initial microbial adhesive interactions–its mechanisms and methods for study. FEMS Microbiol. Rev..

[38] Laverty G., Gorman S.P., Gilmore B.F. (2014). Biomolecular mechanisms of *Pseudomonas aeruginosa* and *Escherichia coli* biofilm formation. Pathogens.

[39] Limoli D.H., Jones C.J., Wozniak D.J. (2015). Bacterial extracellular polysaccharides in biofilm formation and function. Microbiol. Spectr..

[40] Flemming H.C., Neu T.R., Wozniak D.J. (2007). The EPS matrix: The “house of biofilm cells”. J. Bacteriol..

[41] Dufour D., Leung V., Lévesque C.M. (2012). Bacterial biofilm: Structure, function, and antimicrobial resistance. Endod. Top..

[42] Srey S., Jahid I.K., Ha S.D. (2013). Biofilm formation in food industries: A food safety concern. Food Control.

[43] Chatterjee N., Walker G.C. (2017). Mechanisms of DNA damage, repair, and mutagenesis. Environ. Mol. Mutagen..

[44] Greinert R., Volkmer B., Henning S., Breitbart E.W., Greulich K.O., Cardoso M.C., Rapp A. (2012). UVA-induced DNA double-strand breaks result from the repair of clustered oxidative DNA damages. Nucleic Acids Res..

[45] Weiss R.S., Graindorge D., Martineau S., Machon C., Arnoux P., Guitton J., Francesconi S., Frochot C., Sage E., Girard P.M. (2015). Singlet oxygen-mediated oxidation during UVA radiation alters the dynamic of genomic DNA replication. PLoS ONE.

[46] Sorg O., Tran C., Carraux P., Grand D., Hugin A., Didierjean L., Saurat J.H. (2005). Spectral properties of topical retinoids prevent DNA damage and apoptosis after acute UV-B exposure in hairless mice. Photochem. Photobiol..

[47] Kiefer J., Obe G., Natarajan A.T. (2007). Effects of ultraviolet radiation on DNA. Chromosomal Alterations.

[48] Elasri M.O., Miller R.V. (1998). A *Pseudomonas aeruginosa* biosensor responds to exposure to ultraviolet radiation. Appl. Microbiol. Biotechnol..

[49] Elasri M.O., Miller R.V. (1999). Study of the response of a biofilm bacterial community to UV radiation. Appl. Environ. Microb..

[50] Bernbom N., Vogel B.F., Gram L. (2011). *Listeria monocytogenes* survival of UV-C radiation is enhanced by presence of sodium chloride, organic food material and by bacterial biofilm formation. Int. J. Food Microbiol..

[51] Enyedi N.T., Anda D., Borsodi A.K., Szabó A., Pál S.E., Óvári M., Márialigeti K., Kovács-Bodor P., Mádl-Szőnyi J., Makk J. (2019). Radioactive environment adapted bacterial communities constituting the biofilms of hydrothermal spring caves (Budapest, Hungary). J. Environ. Radioact..

[52] Makarova K.S., Omelchenko M.V., Gaidamakova E.K., Matrosova V.Y., Vasilenko A., Zhai M., Lapidus A., Copeland A., Kim E., Land M. (2007). *Deinococcus geothermalis*: The pool of extreme radiation resistance genes shrinks. PLoS ONE.

[53] Frosler J., Panitz C., Wingender J., Flemming H.C., Rettberg P. (2017). Survival of *Deinococcus geothermalis* in biofilms under desiccation and simulated space and martian conditions. Astrobiology.

[54] Cihan A.C., Karaca B., Ozel B.P., Kilic T. (2017). Determination of the bioflm production capacities and characteristics of members belonging to *Bacillaceae* family. World J. Microbiol. Biotechnol..

[55] Inskeep W.P., Macur R.E., Harrison G., Bostick B.C., Fendorf S. (2004). Biomineralization of as (V)-hydrous ferric oxyhydroxide in microbial mats of an acid-sulfate-chloride geothermal spring, Yellowstone National Park. Geochim. Cosmochim. Acta.

[56] Macur R.E., Langner H.W., Kocar B.D., Inskeep W.P. (2004). Linking geochemical processes with microbial community analysis: Successional dynamics in an arsenic-rich, acid-sulphate-chloride geothermal spring. Geobiology.

[57] Kelley J.I., Turng B.F., Williams H.N., Baer M.L. (1997). Effects of temperature, salinity, and substrate on the colonization of surfaces in situ by aquatic bdellovibrios. Appl. Environ. Microbiol..

[58] Williams H.N., Turng B.F., Kelley J.I. (2009). Survival response of *Bacteriovorax* in surface biofilm versus suspension when stressed by extremes in environmental conditions. Microb. Ecol..

[59] Caruso C., Rizzo C., Mangano S., Poli A., Donato P.D., Finore I., Nicolaus B., Marco G.D., Michaud L., Giudice A.L. (2018). Production and biotechnological potential of extracellular polymeric substances from sponge-associated antarctic bacteria. Appl. Environ. Microb..

[60] Hujslova M., Bystriansky L., Benada O., Gryndler M. (2019). Fungi, a neglected component of acidophilic biofilms: Do they have a potential for biotechnology?. Extremophiles.

[61] Li T., Sharp C.E., Ataeian M., Strous M., de Beer D. (2018). Role of extracellular carbonic anhydrase in dissolved inorganic carbon uptake in alkaliphilic phototrophic biofilm. Front. Microbiol..

[62] Li X., Kappler U., Jiang G., Bond P.L. (2017). The ecology of acidophilic microorganisms in the corroding concrete sewer environment. Front. Microbiol..

[63] Bellenberg S., Huynh D., Poetsch A., Sand W., Vera M. (2019). Proteomics reveal enhanced oxidative stress responses and metabolic adaptation in *Acidithiobacillus ferrooxidans* biofilm cells on pyrite. Front. Microbiol..

[64] Aguilera A., Souza-Egipsy V., Martín-Uriz P.S., Amils R. (2008). Extracellular matrix assembly in extreme acidic eukaryotic biofilms and their possible implications in heavy metal adsorption. Aquat. Toxicol..

[65] Hawkins P.T., Poyner D.R., Jackson T.R., Letcher A.J., Lander D.A., Irvine R.F. (1993). Inhibition of iron-catalysed hydroxyl radical formation by inositol polyphosphates: A possible physiological function for *myo*-inositol hexakisphosphate. Biochem. J..

[66] Shah V., Ray A., Garg N., Madamwar D. (2000). Characterization of the extracellular polysaccharide produced by a marine cyanobacterium, *Cyanothece* sp. ATCC 51142, and its exploitation toward metal removal from solutions. Curr. Microbiol..

[67] Charles C.J., Rout S.P., Garratt E.J., Patel K., Laws A.P., Humphreys P.N. (2015). The enrichment of an alkaliphilic biofilm consortia capable of the anaerobic degradation of isosaccharinic acid from cellulosic materials incubated within an anthropogenic, hyperalkaline environment. FEMS Microbiol. Ecol..

[68] Rout S.P., Payne L., Walker S., Scott T., Heard P., Eccles H., Bond G., Shah P., Bills P., Jackson B.R. (2018). The impact of alkaliphilic biofilm formation on the release and retention of carbon isotopes from nuclear reactor graphite. Sci. Rep..

[69] Charles C.J., Rout S.P., Patel K.A., Akbar S., Laws A.P., Jackson B.R., Boxall S.A., Humphreys P.N. (2017). Floc formation reduces the pH stress experienced by microorganisms living in alkaline environments. Appl. Environ. Microbiol..

[70] Chávez de Paz L.E., Bergenholtz G., Dahlén G., Svensäter G. (2007). Response to alkaline stress by root canal bacteria in biofilms. Int. Endod. J..

[71] Van der Waal S.V., van der Sluis L.W., Özok A.R., Exterkate R.A., van Marle J., Wesselink P.R., de Soet J.J. (2011). The effects of hyperosmosis or high pH on a dual-species biofilm of *Enterococcus faecalis* and *Pseudomonas aeruginosa*: An in vitro study. Int. Endod. J..

[72] Vyrides I., Stuckey D.C. (2009). Adaptation of anaerobic biomass to saline conditions: Role of compatible solutes and extracellular polysaccharides. Enzyme Microb. Technol..

[73] Kimata-Kino N., Ikeda S., Kurosawa N., Toda T. (2011). Saline adaptation of granules in mesophilic UASB reactors. Int. Biodeter. Biodegr..

[74] Gagliano M.C., Ismail S.B., Stams A.J.M., Plugge C.M., Temmink H., Van Lier J.B. (2017). Biofilm formation and granule properties in anaerobic digestion at high salinity. Water Res..

[75] Adamiak J., Otlewska A., Gutarowska B. (2015). Halophilic microbial communities in deteriorated buildings. World J. Microb. Biot..

[76] Amjres H., Bejar V., Quesada E., Carranza D., Abrini J., Sinquin C., Ratiskol J., Colliec-Jouault S., Llamas I. (2015). Characterization of haloglycan, an exopolysaccharide produced by *Halomonas stenophila* HK30. Int. J. Biol. Macromol..

[77] Mallick I., Bhattacharyya C., Mukherji S., Dey D., Sarkar S.C., Mukhopadhyay U.K., Ghosh A. (2018). Effective rhizoinoculation and biofilm formation by arsenic immobilizing halophilic plant growth promoting bacteria (PGPB) isolated from mangrove rhizosphere: A step towards arsenic rhizoremediation. Sci. Total Environ..

[78] Zhao L., She Z., Jin C., Yang S., Guo L., Zhao Y., Gao M. (2016). Characteristics of extracellular polymeric substances from sludge and biofilm in a simultaneous nitrification and denitrification system under high salinity stress. Bioprocess Biosyst. Eng..

[79] You G., Hou J., Xu Y., Wang C., Wang P., Miao L., Ao Y., Li Y., Lv B. (2015). Effects of CeO_2_ nanoparticles on production and physicochemical characteristics of extracellular polymeric substances in biofilms in sequencing batch biofilm reactor. Bioresour. Technol..

[80] Kato C., Qureshi M.H. (1999). Pressure response in deep-sea piezophilic bacteria. J. Molec. Microbiol. Biotechnol..

[81] Simonato F., Campanaro S., Lauro F.M., Vezzi A., D’Angelo M., Vitulo N., Valle G., Bartlett D.H. (2006). Piezophilic adaptation: A genomic point of view. J. Biotechnol..

[82] Masanta W.O., Hinz R., Zautner A.E. (2015). Infectious causes of cholesteatoma and treatment of infected ossicles prior to reimplantation by hydrostatic high-pressure inactivation. BioMed Res. Int..

[83] Pavlovic M., Hormann S., Vogel R.F., Ehrmann M.A. (2005). Transcriptional response reveals translation machinery as target for high pressure in *Lactobacillus sanfranciscensis*. Arch. Microbiol..

[84] Kadam S.R., den Besten H.M., van der Veen S., Zwietering M.H., Moezelaar R., Abee T. (2013). Diversity assessment of *Listeria monocytogenes* biofilm formation: Impact of growth condition, serotype and strain origin. Int. J. Food Microbiol..

[85] Combrouse T., Sadovskaya I., Faille C., Kol O., Guerardel Y., Midelet-Bourdin G. (2013). Quantification of the extracellular matrix of the *Listeria monocytogenes* biofilms of different phylogenic lineages with optimization of culture conditions. J. Appl. Microbiol..

[86] Cherifi T., Jacques M., Quessy S., Fravalo P. (2017). Impact of nutrient restriction on the structure of *Listeria monocytogenes* biofilm grown in a microfluidic system. Front. Microbiol..

[87] Smith D.R., Maestre-Reyna M., Lee G., Gerard H., Wang A.H., Watnick P.I. (2015). In situ proteolysis of the *Vibrio cholerae* matrix protein RbmA promotes biofilm recruitment. Proc. Natl. Acad. Sci. USA.

[88] Fong J.C., Yildiz F.H. (2008). Interplay between cyclic AMP-cyclic AMP receptor protein and cyclic di-GMP signaling in *Vibrio cholerae* biofilm formation. J. Bacteriol..

[89] Falkinham J.O. (2009). Surrounded by mycobacteria: Nontuberculous mycobacteria in the human environment. J. Appl. Microbiol..

[90] Mittelman M.W., Jones A.D.G. (2018). A pure life: The microbial ecology of high purity industrial waters. Microb. Ecol..

[91] Olsen I. (2015). Biofilm-specific antibiotic tolerance and resistance. Eur. J. Clin. Microbiol..

[92] Blair J.M., Webber M.A., Baylay A.J., Ogbolu D.O., Piddock L.J. (2015). Molecular mechanisms of antibiotic resistance. Nat. Rev. Microbiol..

[93] Anderl J.N., Franklin M.J., Stewart P.S. (2000). Role of antibiotic penetration limitation in *Klebsiella pneumoniae* biofilm resistance to ampicillin and ciprofloxacin. Antimicrob. Agents Chemother..

[94] Dunne W.M., Mason E.O., Kplan S.L. (1993). Diffusion of rifampin and vancomycin through a *Staphylococcus epidermidis* biofilm. Antimicrob. Agents Chemother..

[95] Nadell C.D., Drescher K., Wingreen N.S., Bassler B.L. (2015). Extracellular matrix structure governs invasion resistance in bacterial biofilms. ISME J..

[96] De la Fuente-Núñez C., Reffuveille F., Fernández L., Hancock R.E. (2013). Bacterial biofilm development as a multicellular adaptation: Antibiotic resistance and new therapeutic strategies. Curr. Opin. Microbiol..

[97] Singh R., Ray P., Das A., Sharma M. (2010). Penetration of antibiotics through *Staphylococcus aureus* and *Staphylococcus epidermidis* biofilms. J. Antimicrob. Chemother..

[98] Kumon H., Tomochika K., Matunaga T., Ogawa M., Ohmoril H. (1994). A sandwich cup method for the penetration assay of antimicrobial agents through *Pseudomonas* exopolysaccharides. Microbiol. Immunol..

[99] Giwercman B., Jensen E.T., Høiby N., Kharazmi A., Costerton J.W. (1991). Induction of β-lactamase production in *Pseudomonas aeruginosa* biofilm. Antimicrob. Agents Chemother..

[100] Stewart P.S. (1996). Theoretical aspects of antibiotic diffusion into microbial biofilms. Antimicrob. Agents Chemother..

[101] Jennings L.K., Storek K.M., Ledvina H.E., Coulon C., Marmont L.S., Sadovskaya I., Secor P.R., Tseng B.S., Scian M., Filloux A. (2015). Pel is a cationic exopolysaccharide that cross-links extracellular DNA in the *Pseudomonas aeruginosa* biofilm matrix. Proc. Natl. Acad. Sci. USA.

[102] Colvin K.M., Gordon V.D., Murakami K., Borlee B.R., Wozniak D.J., Wong G.C., Parsek M.R. (2011). The Pel polysaccharide can serve a structural and protective role in the biofilm matrix of *Pseudomonas aeruginosa*. PLoS Pathog..

[103] Dogtrid I.G., Evans E., Brown M.R.W., Gilbert P. (1992). Growth-rate-independent killing by ciprofloxacin of biofilm-derived *Staphylococcus epidermidis* evidence for cell-cycle dependency. J. Antimicrob. Chemother..

[104] Evans D.J., Allison D.G., Brown M.R.W., Gilbert P. (1991). Susceptibility of *Pseudomonas aeruginosa* and *Escherichia coli* biofilms towards ciprofloxacin: Effect of specific growth rate. J. Antimicrob. Chemother..

[105] Gilbert P., Collier P.J., Brown M.R.W. (1990). Influence of growth rate on susceptibility to antimicrobial agents: Biofilms, cell cycle, dormancy, and stringent response. Antimicrob. Agents Chemother..

[106] Anwar H., Strap J.L., Costerton J.W. (1992). Establishment of aging biofilms: Possible mechanism of bacterial resistance to antimicrobial therapy. Antimicrob. Agents Chemother..

[107] Mcleod G.I., Spector M.P. (1996). Starvation- and stationary-phase-induced resistance to the antimicrobial peptide polymyxin B in *Salmonella typhimurium* is RpoS (σ^S^) independent and occurs through both *pho*P-dependent and -independent pathway. J. Bacteriol..

[108] Williamson K.S., Richards L.A., Perez-Osorio A.C., Pitts B., McInnerney K., Stewart P.S., Franklin M.J. (2012). Heterogeneity in *Pseudomonas aeruginosa* biofilms includes expression of ribosome hibernation factors in the antibiotic-tolerant subpopulation and hypoxia-induced stress response in the metabolically active population. J. Bacteriol..

[109] Lewis K. (2012). Persister cells: Molecular mechanisms related to antibiotic tolerance. Handb. Exp. Pharmacol..

[110] Mah T.F., Pitts B., Pellock B., Walker G.C., Stewart P.S., O’Toole G.A. (2003). A genetic basis for *Pseudomonas aeruginosa* biofilm antibiotic resistance. Nature.

[111] Bae J., Oh E., Jeon B. (2014). Enhanced transmission of antibiotic resistance in *Campylobacter jejuni* biofilms by natural transformation. Antimicrob. Agents Chemother..

[112] Limoli D.H., Rockel A.B., Host K.M., Jha A., Kopp B.T., Hollis T., Wozniak D.J. (2014). Cationic antimicrobial peptides promote microbial mutagenesis and pathoadaptation in chronic infections. PLoS Pathog..

[113] Cook L.C., Dunny G.M. (2013). Effects of biofilm growth on plasmid copy number and expression of antibiotic resistance genes in *Enterococcus faecalis*. Antimicrob. Agents Chemother..

[114] Poole K. (2001). Multidrug resistance in Gram-negative bacteria. Curr. Opin. Microbiol..

[115] Sun J., Deng Z., Yan A. (2014). Bacterial multidrug efflux pumps: Mechanisms, physiology and pharmacological exploitations. Biochem. Biophys. Res. Commun..

[116] Fraud S., Poole K. (2011). Oxidative stress induction of the MexXY multidrug efflux genes and promotion of aminoglycoside resistance development in *Pseudomonas aeruginosa*. Antimicrob. Agents Chemother..

[117] Zhang L., Mah T.F. (2008). Involvement of a novel efflux system in biofilm-specific resistance to antibiotics. J. Bacteriol..

[118] Wang Q., Sun F.J., Liu Y., Xiong L.R., Xie L.L., Xia P.Y. (2010). Enhancement of biofilm formation by subinhibitory concentrations of macrolides in icaADBC-positive and -negative clinical isolates of *Staphylococcus epidermidis*. Antimicrob. Agents Chemother..

[119] Rachid S., Ohlsen K., Witte W., Hacker J.R., Ziebuhr W. (2000). Effect of subinhibitory antibiotic concentrations on polysaccharide intercellular adhesin expression in biofilm-forming *Staphylococcus epidermidis*. Antimicrob. Agents Chemother..

[120] Wu S., Li X., Gunawardana M., Maguire K., Guerrero-Given D., Schaudinn C., Wang C., Baum M.M., Webster P. (2014). Beta-lactam antibiotics stimulate biofilm formation in non-typeable *Haemophilus influenzae* by up-regulating carbohydrate metabolism. PLoS ONE.

[121] Burmolle M., Ren D., Bjarnsholt T., Sorensen S.J. (2014). Interactions in multispecies biofilms: Do they actually matter?. Trends Microbiol..

[122] Roder H.L., Sorensen S.J., Burmolle M. (2016). Studying bacterial multispecies biofilms: Where to start?. Trends Microbiol..

[123] Burmolle M., Webb J.S., Rao D., Hansen L.H., Sorensen S.J., Kjelleberg S. (2006). Enhanced biofilm formation and increased resistance to antimicrobial agents and bacterial invasion are caused by synergistic interactions in multispecies biofilms. Appl. Environ. Microbiol..

[124] De Brucker K., Tan Y., Vints K., De Cremer K., Braem A., Verstraeten N., Michiels J., Vleugels J., Cammue B.P., Thevissen K. (2015). Fungal beta-1,3-glucan increases ofloxacin tolerance of *Escherichia coli* in a polymicrobial *E. coli*/*Candida albicans* biofilm. Antimicrob. Agents Chemother..

[125] Shirtliff M.E., Peters B.M., Jabra-Rizk M.A. (2009). Cross-kingdom interactions: *Candida albicans* and bacteria. FEMS Microbiol. Lett..

[126] Harriott M.M., Noverr M.C. (2009). *Candida albicans* and *Staphylococcus aureus* form polymicrobial biofilms: Effects on antimicrobial resistance. Antimicrob. Agents Chemother..

[127] Peters B.M., Jabra-Rizk M.A., Scheper M.A., Leid J.G., Costerton J.W., Shirtliff M.E. (2010). Microbial interactions and differential protein expression in *Staphylococcus aureus*-*Candida albicans* dual-species biofilms. FEMS Immunol. Med. Microbiol..

[128] Gambino M., Cappitelli F. (2016). Mini-review: Biofilm responses to oxidative stress. Biofouling.

[129] Matin A., Lynch S.V. (2005). Investigating the threat of bacteria grown in space. ASM News.

[130] Montgomery K., Charlesworth J.C., LeBard R., Visscher P.T., Burns B.P. (2013). Quorum sensing in extreme environments. Life.

[131] Papenfort K., Bassler B.L. (2016). Quorum sensing signal-response systems in Gram-negative bacteria. Nat. Rev. Microbiol..

[132] Moradali M.F., Ghods S., Rehm B.H. (2017). *Pseudomonas aeruginosa* Lifestyle: A paradigm for adaptation, survival, and persistence. Front. Cell Infect. Microbiol..

[133] Shih P.C., Huang C.T. (2002). Effects of quorum-sensing deficiency on *Pseudomonas aeruginosa* biofilm formation and antibiotic resistance. J. Antimicrob. Chemother..

[134] Bjarnsholt T., Jensen P.O., Burmolle M., Hentzer M., Haagensen J.A., Hougen H.P., Calum H., Madsen K.G., Moser C., Molin S. (2005). *Pseudomonas aeruginosa* tolerance to tobramycin, hydrogen peroxide and polymorphonuclear leukocytes is quorum-sensing dependent. Microbiol. -SGM.

[135] Ciofu O., Tolker-Nielsen T., Jensen P.O., Wang H., Hoiby N. (2015). Antimicrobial resistance, respiratory tract infections and role of biofilms in lung infections in cystic fibrosis patients. Adv. Drug Deliv. Rev..

[136] Zhu J., Mekalanos J.J. (2003). Quorum sensing-dependent biofilms enhance colonization in *Vibrio cholerae*. Dev. Cell.

[137] March J.C., Bentley W.E. (2004). Quorum sensing and bacterial cross-talk in biotechnology. Curr. Opin. Biotechnol..

[138] Bhargava N., Sharma P., Capalash N. (2012). N-acyl homoserine lactone mediated interspecies interactions between *A. baumannii* and *P. aeruginosa*. Biofouling.

[139] Sun Z., He X., Brancaccio V.F., Yuan J., Riedel C.U. (2014). Bifidobacteria exhibit LuxS-dependent autoinducer 2 activity and biofilm formation. PLoS ONE.

[140] Díaz M., Castro M., Copaja S., Guiliani N. (2018). Biofilm formation by the acidophile bacterium *Acidithiobacillus thiooxidans* involves c-di-GMP pathway and Pel exopolysaccharide. Genes.

[141] Huynh T.T., McDougald D., Klebensberger J., Al Qarni B., Barraud N., Rice S.A., Kjelleberg S., Schleheck D. (2012). Glucose starvation-induced dispersal of *Pseudomonas aeruginosa* biofilms is cAMP and energy dependent. PLoS ONE.

[142] Galloway W.R.J.D., Hodgkinson J.T., Bowden S.D., Welch M., Spring D.R. (2011). Quorum sensing in Gram-negative bacteria: Small-molecule modulation of AHL and AI-2 quorum sensing pathways. Chem. Rev..

[143] Lin L., Dai S., Tian B., Li T., Yu J., Liu C., Wang L., Xu H., Zhao Y., Hua Y. (2016). DqsIR quorum sensing-mediated gene regulation of the extremophilic bacterium *Deinococcus radiodurans* in response to oxidative stress. Mol. Microbiol..

[144] Mamani S., Moinier D., Denis Y., Soulere L., Queneau Y., Talla E., Bonnefoy V., Guiliani N. (2016). Insights into the quorum sensing regulon of the acidophilic *Acidithiobacillus ferrooxidans* revealed by transcriptomic in the presence of an Acyl homoserine lactone superagonist analog. Front. Microbiol..

[145] Moya-Beltran A., Rojas-Villalobos C., Diaz M., Guiliani N., Quatrini R., Castro M. (2019). Nucleotide second messenger-based signaling in extreme acidophiles of the *Acidithiobacillus* species complex: Partition between the core and variable gene complements. Front. Microbiol..

[146] Coggan K.A., Wolfgang M.C. (2012). Global regulatory pathways and cross-talk control *Pseudomonas aeruginosa* environmental lifestyle and virulence phenotype. Curr. Issues Mol. Biol..

[147] Hengge R. (2009). Principles of c-di-GMP signalling in bacteria. Nat. Rev. Microbiol..

[148] Römling U., Amikam D. (2006). Cyclic di-GMP as a second messenger. Curr. Opin. Microbiol..

[149] Irie Y., Borlee B.R., O’Connor J.R., Hill P.J., Harwood C.S., Wozniak D.J., Parsek M.R. (2012). Self-produced exopolysaccharide is a signal that stimulates biofilm formation in *Pseudomonas aeruginosa*. Proc. Natl. Acad. Sci. USA.

[150] Jain R., Behrens A.J., Kaever V., Kazmierczak B.I. (2012). Type IV pilus assembly in *Pseudomonas aeruginosa* over a broad range of cyclic di-GMP concentrations. J. Bacteriol..

[151] Ueda A., Wood T.K. (2010). Tyrosine phosphatase TpbA of *Pseudomonas aeruginosa* controls extracellular DNA via cyclic diguanylic acid concentrations. Environ. Microbiol. Rep..

[152] Tischler A.D., Camilli A. (2004). Cyclic diguanylate (c-di-GMP) regulates *Vibrio cholerae* biofilm formation. Mol. Microbiol..

[153] O’Toole G.A., Kolter R. (1998). Flagellar and twitching motility are necessary for *Pseudomonas aeruginosa* biofilm development. Mol. Microbiol..

[154] Semmler A.B.T., Whitchurch C.B., Mattick J.S. (1999). A re-examination of twitching motility in *Pseudomonas aeruginosa*. Microbiol. -SGM.

[155] Paytubi S., Cansado C., Madrid C., Balsalobre C. (2017). Nutrient composition promotes switching between pellicle and bottom biofilm in *Salmonella*. Front. Microbiol..

[156] Toyofuku M., Inaba T., Kiyokawa T., Obana N., Yawata Y., Nomura N. (2016). Environmental factors that shape biofilm formation. Biosci. Biotechnol. Biochem..

